# Indicators of Suboptimal Treatment and Associated Healthcare Costs Among Patients With Crohn’s Disease Initiated on Biologic or Conventional Agents

**DOI:** 10.1093/crocol/otac021

**Published:** 2022-06-16

**Authors:** Dominic Pilon, Zhijie Ding, Erik Muser, Ameur M Manceur, Maude Vermette-Laforme, Marie-Hélène Lafeuille, Patrick Lefebvre

**Affiliations:** Analysis Group, Inc., Montreal, Quebec, Canada; Janssen Scientific Affairs, LLC, Horsham, Pennsylvania, USA; Janssen Scientific Affairs, LLC, Horsham, Pennsylvania, USA; Analysis Group, Inc., Montreal, Quebec, Canada; Analysis Group, Inc., Montreal, Quebec, Canada; Analysis Group, Inc., Montreal, Quebec, Canada; Analysis Group, Inc., Montreal, Quebec, Canada

**Keywords:** Crohn’s disease, suboptimal treatment, healthcare costs, biologic agents, conventional agents, burden

## Abstract

**Background:**

As the treatment landscape for Crohn’s disease (CD) evolves, an up-to-date understanding of the burden associated with indicators of suboptimal treatment is needed. The aim of this study was to describe suboptimal treatment indicators and associated healthcare costs among CD patients initiated on a biologic or conventional agent.

**Methods:**

Adults with CD were identified in a US healthcare claims database (Optum’s Clinformatics Data Mart; 01/2004–03/2019). The first biologic or conventional agent claim within 12 months of a CD diagnosis was the index date/agent. Indicators of suboptimal treatment (nonadherence, dose escalation, chronic corticosteroid use, augmentation, ≥1 CD surgery, ≥2 CD emergency department visits, ≥1 CD inpatient (IP) stay, switch, cycling, restart, inadequate induction) were identified in the 12-month postindex landmark period. The mean per-patient-per-year (PPPY) healthcare costs (2019 USD) were evaluated in the year postlandmark.

**Results:**

There were 5107 patients (mean age ~44 years, 56% female) in the biologic and 6072 patients (~51 years; 59% female) in the conventional cohort. In the biologic cohort, 79.4% of patients had ≥1 suboptimal treatment indicator. Mean PPPY healthcare costs increased with the number of suboptimal treatment indicators, from $46 100 (no indicator) to $68 572 (≥4 indicators). The conventional cohort had similar patterns: 72.5% of patients presented ≥1 suboptimal treatment indicator, and mean PPPY healthcare costs increased from $17 329 (no indicator) to $67 568 (≥4 indicators). In both cohorts, IP and outpatient medical costs (excluding biologics) contributed a major portion of the increase.

**Conclusions:**

Among CD patients, suboptimal treatment indicators were common and were associated with an increased burden to the healthcare system.

## Introduction

Crohn’s disease (CD) is a subtype of inflammatory bowel disease (IBD) that may affect as many as 780 000 persons in the United States^1^ and that is associated with an important economic burden to the healthcare system and to society.^2–5^ As opposed to ulcerative colitis (UC) that is limited to the colon, CD can occur in any part of the digestive system from the mouth to the anus and can affect all layers of the bowel walls.^6,7^ CD is associated with physical symptoms such as abdominal pain, diarrhea, fatigue, weight loss, and blood in stools, in addition to an important quality-of-life burden and a reduction in workplace and household productivity.^6–8^ CD, and its concomitant cumulative damage, can also lead to more severe intestinal complications over time such as bowel obstruction, ulcers, and fistula.^6,7^

Several treatments are available to control the symptoms of CD and to achieve and maintain remission. Depending on the severity of the disease, conventional therapy including 5-aminosalicylate [5-ASA] agents (eg, mesalamine) and immunomodulators (eg, azathioprine) to maintain remission are frequently used first, despite evidence demonstrating the lack of efficacy of 5-ASA agents.^7,9^ Corticosteroids are associated with undesired adverse events and are generally used only for short-term treatment.^7,9^ Based on the response, notably steroid-dependent/refractory disease and an evaluation of risk for disease progression, biologic agents are then used.^7,9^ Over the last 2 decades these agents have grown in importance and different mechanisms of action (ie, antitumor necrosis factor, anti-integrin, and anti-interleukin) are now available.^10^ If medications do not provide sufficient response, surgical treatment (eg, segmental resection, proctocolectomy, and colectomy) is evaluated along with maintenance medication.^9,10^

Approaches to optimize the use of the various medications available and reduce the proportion of treatment failure have been discussed in the literature using mostly information from clinical trials.^11,12^ For instance, if the first biologic tried does not provide sufficient response, optimization by dose escalation, combination therapy with an immunomodulator, or switch to a different biologic or class of biologic are recommended.^11^ For conventional agents, in case treatment objectives (eg, corticosteroid-free remission, reduced hospital admission) are not achieved, measures that improve adherence, treatment switch, dose escalation, and metabolite monitoring to tailor medication have been recommended.^12^

In the real world, a systematic review of 41 studies covering the years 2012–2017 concluded that biologic use among patients with IBD in the Unites States may be suboptimal with high but variable rates of nonadherence (23%–62%), discontinuation (7%–65%), switch to another biologic (5%–20%), and dose escalation (8%–35%).^13^ Similarly, a claim-based study by Rubin et al covering the years 2006–2010 concluded that among patients with CD treated with 5-ASA or immunomodulators in the United States, indicators of suboptimal treatment, such as discontinuation or augmentation with another agent, were common.^14^ Both of these studies also reported that patients with a suboptimal therapy indicator experienced higher healthcare costs compared to patients without indicator of suboptimal therapy.^13,14^

As the treatment landscape for CD has continued to evolve, notably with the approval of vedolizumab in 2014^15^ and of ustekinumab in 2016,^16^ it is important for healthcare stakeholders to have a comprehensive, up-to-date understanding of treatment patterns and the extent of the economic burden associated with suboptimal treatment indicators. The objective of the study was to describe indicators of suboptimal treatment and associated healthcare costs among patients with CD initiated on a biologic or a conventional agent.

## Methods

### Data Source

This study was conducted using the Optum’s Clinformatics Data Mart database spanning a 15-year period from 01/2004 through 03/2019. This database included medical and prescription drug claims, insurance eligibility, and demographics for approximately 57 million privately insured individuals in the United States. This database is deidentified and complies with the patient confidentiality requirements of the Health Insurance Portability and Accountability Act (HIPAA).

### Study Design

A retrospective longitudinal cohort design was used to describe cohorts of patients using biologic agents (biologic cohort) and conventional agents (conventional cohort). The index date was defined as the date of the first claim for an agent of interest (index biologic or conventional agent) on the date of or ≤12 months after a diagnosis for CD (International Classification of Diseases, version 9 [ICD-9] code 555.x or International Classification of Diseases, version 10 [ICD-10] code K50.x; Supplementary Figure S1). Cohorts were not mutually exclusive, for example a patient could be part of both the conventional and biologic cohorts with different index dates and agents. However, patients with a claim for both a biologic and conventional agent on the index date only contributed to the biologic cohort.

The biologic agents included tumor necrosis factor inhibitors (ie, adalimumab, infliximab [including biosimilars], and certolizumab), anti-integrin agents (ie, natalizumab and vedolizumab), and anti-interleukin agents (ie, ustekinumab). The conventional agents included 5-ASA agents (ie, mesalamine, sulfasalazine, balsalazide, and olsalazine), and immunomodulators (ie, azathioprine, mercaptopurine, methotrexate, mycophenolate, tacrolimus, cyclosporine, leflunomide, and thalidomide). Given that corticosteroids are often used as a bridge to another therapy (ie, for short-term use to treat symptoms of flares between 2 longer-term therapies), initiation of a corticosteroid was not considered as an independent conventional therapy line.^14,17^

Patient characteristics were described during the 12-month baseline period prior to the index date. A 12-month postindex landmark period was used to describe treatment patterns and classify patients in subgroups based on indicators of suboptimal treatment (Table 1). Healthcare costs were evaluated during the 12-month period following the end of the landmark year (follow-up period) and stratified by each suboptimal treatment indicator and by number of indicators.

**Table 1.** Indicators of suboptimal biological or conventional treatment among patients with CD during the landmark year (12 months following initiation of index agent).

Biologic and conventional cohorts	
Nonadherence	Among patients with at least 2 claims for the index agent, medication possession ratio (MPR) <80% (MPR: sum of index agent days of supply divided by the number of days between the first and last day of supply of the index agent within the landmark period)
Dose escalation^a^	*Biologic*: A reference daily exposure,^b^^,^^c^ to the index agent was calculated during the 90 days after the index date. Dose escalation occurred when the daily exposure after the 90-day period was 50% above the reference daily exposure for a fill date
	*Conventional*: A reference daily exposure^b^ to the index agent was calculated during the 90 days (immunomodulators) or 45 days (5-ASA) after the index date. Dose escalation occurred when the daily exposure was 10% (immunomodulators) or 50% (5-ASA) above the reference daily exposure for a fill date; for mesalamine dose escalation also occurred with combination therapy oral + rectal independently from the dose
Restart	Among patients with discontinuation of the index agent (ie, 90 days gap in the day of supply), ≥1 fill for the index medication between the discontinuation date and the end of the landmark year
Chronic corticosteroid use	≥90 days of nonoverlapping days of supply of corticosteroids cumulated over the landmark year
Augmentation^a^	*Biologic*: To exclude combination therapies, in the 30 days following the index date (buffer period), immunomodulators use was recorded and these agents could *not* be candidate for augmentation. Following the buffer period, augmentation was defined as ≥60 days concomitant use of an immunomodulator candidate for augmentation
	*Conventional*: ≥60 days of concomitant use of a biologic candidate for augmentation following a buffer period of 30 days after the index date
≥1 surgery for CD, ≥2 ED visits for CD, ≥1 IP for CD	Defined based on medical claims with an ICD-9 diagnosis of 555.x or an ICD-10 diagnosis of K50.x during the landmark year
Biologic cohort	
Switch to conventional	Among patients with discontinuation of the index biologic (ie, 90 days gap in the day of supply), ≥1 fill for a conventional agent (ie, 5-ASA or immunomodulator) between the discontinuation date and the end of the landmark year
Switch to a different biologic	Among patients with discontinuation of the index biologic (ie, 90 days gap in the day of supply), ≥1 fill for a biologic agent other than the index biologic between the discontinuation date and the end of the landmark year
Inadequate induction	Depending on the index agent, either the number of fills (infliximab, vedolizumab) or the total dose (adalimumab, certolizumab) in the induction period per label with 25% grace period (eg, 6 weeks + 1.5 week buffer for infliximab) was counted. Inadequate induction dose occurred when the number of fills was fewer than expected per label (eg, <3 fills for infliximab or <240 mg for adalimumab) during that period. For ustekinumab, inadequate induction occurred if the record of use on the index date was not for an intravenous administration. For natalizumab, the indicator could not be estimated as induction is not indicated
Conventional cohort	
Switch from index treatment	Among patients with discontinuation of the index conventional (ie, 90 days gap in the day of supply), ≥1 fill for an agent different than the index agent mechanism of action (including both conventional and biologic agents) between the discontinuation date and the end of the landmark year
Cycling on index treatment mechanism of action	Among patients with discontinuation of the index conventional (ie, 90 days gap in the day of supply), ≥1 fill for an agent in the index treatment mechanism of action (ie, 5-ASA or immunomodulators, excluding the index agent) between the discontinuation date and the end of the landmark year

Abbreviations: 5-ASA, 5-aminosalicylic acid; CD, Crohn’s disease; ED, emergency department; ICD-9, International Classification of Diseases, version 9; ICD-10, International Classification of Diseases, version 10; IP, inpatient.

Evaluated during the first continuous episode of use of the index therapy (ie, without a gap of ≥90 consecutive days between days of index treatment supply or between the last day of supply and the end of the landmark period). If there was no discontinuation, the first continuous episode of use of the index therapy was censored at the end of the landmark year.

In the pharmacy claims, the ratio of the total dose and days of supply at a claim was used to estimate the daily exposure, and in the medical claims the ratio of the number of units and time interval between claims was used to estimate the daily exposure. The weight of the patient was assumed to be constant.

For natalizumab, there was no reference daily exposure as induction is not indicated for this drug (ie, the reference daily dose was calculated based on the index date).

### Study Sample

The study included adult patients (≥18 years old) with ≥12 months of continuous health plan eligibility before and ≥24 months of continuous health plan eligibility after the index date. Patients were required to have ≥2 independent claims with a CD diagnosis ≥30 days apart in the 12-month period preceding the index date. Given that UC and CD are related, patients were classified as having CD using an algorithm based on the frequency of diagnoses.^18,19^ Specifically, patients were kept in the study sample based on the following hierarchy (1) they had more CD-related inpatient (IP) admissions than UC-related IP admissions in the 12 months before the index date, (2) if they had CD-related claims for ≥75% of the total number of CD/UC-related claims in the 12 months before the index date, or (3) if the last 2 CD/UC-related claims in the 12 months before the index date were CD-related claims.

To be included in the biologic cohort, the index agent could not be used in the 12 months before the index date (ie, agent washout), and no other biologic agent than the index agent could be used on the index date. To be included in the conventional cohort, in the 12 months before the index date, the index agent could not be used (ie, agent washout) and no biologic agent could be used; in addition, on the index date, no other conventional agent than the index agent could be used.

### Indicators of Suboptimal Treatment in the Landmark Period

In the study sample, patient subgroups were identified based on indicators of suboptimal treatment evaluated during the landmark period (12-month period following the index date). In both cohorts, nonadherence, dose escalation, restart, chronic corticosteroid use, augmentation, ≥1 surgery for CD, ≥2 emergency department (ED) visits for CD, and ≥1 IP for CD were evaluated (see specific definitions in Table 1). In the biologic cohort, switch to a conventional agent, switch to a different biologic, and inadequate induction were also evaluated. In the conventional cohort, switch from index treatment and cycling on the index treatment mechanism of action were also evaluated. Moreover, in both cohorts, patients without an indicator for suboptimal treatment, and with ≥1, 2, 3, and 4 indicators of suboptimal treatment were identified. Of note, augmentation was distinguished from combination therapy since agents used in the first 30 days following the index date were not candidate for augmentation. Note also that discontinuation alone was not a suboptimal indicator. However, restarts, switches, and cycling following discontinuation were considered suboptimal indicators if these events occurred within 12 months of initiation.

### Outcome Measures in the Follow-up Period

Healthcare costs were evaluated during the 12-month period following the end of the landmark year (follow-up period). Costs were evaluated from the payer perspective and included the total cost, and the following categories: total cost of biologics included in the study (sum of the biologic injection/infusion cost in medical claims and the biologic medication costs in pharmacy claims); medical costs (excluding biologic costs) for IP, ED, outpatient (OP), and other visits; and pharmacy costs (excluding biologic costs).

### Statistical Analysis

Descriptive statistics for the baselines, treatment patterns and healthcare costs were reported as mean with SD for continuous variables and as frequencies with proportion for categorical variables. Healthcare costs were reported as per-patient-per-year (PPPY). Costs were inflated using the medical care component of US Consumer Price Index for Urban Consumers and reported in United States dollars 2019 (USD 2019). SAS Enterprise Guide 7.1 (SAS Institute) was used to conduct all analyses.

## Results

### Baseline Characteristics

#### Biologic cohort

In the biologic cohort, a total of 5107 CD patients initiated on a biologic (mean age 43.9 years, 56.0% female) were included (see Table 2), of whom 80 (1.6%) used both a biologic and an immunomodulator at index. The most common index agent was infliximab (42.7%), followed by adalimumab (36.0%), certolizumab (14.0%), with remaining patients on vedolizumab (5.5%), ustekinumab (1.4%), or natalizumab (0.4%). The mean Quan-Charlson Comorbidity Index (Quan-CCI) was 0.75, and 64.9% of the patients were considered to have claim-derived moderate-to-severe CD. The proportion of patients with ≥1 corticosteroid claim was 72.2%, and ≥1 opioid claim was 59.8%. Finally, the mean baseline PPPY healthcare costs were $34 084.

**Table 2.** Characteristics evaluated during the 12-month baseline period before the initiation of the index agent among patients with CD.

Baseline period	Biologic cohort *N* = 5107	Conventional cohort *N* = 6072
Age, mean ± SD	43.9 ± 15.4	50.5 ± 16.7
Female, *N* (%)	2860 (56.0%)	3604 (59.4%)
Region of residence, *N* (%)		
Northeast	540 (10.6%)	724 (11.9%)
Midwest	1625 (31.8%)	1629 (26.8%)
South	2052 (40.2%)	2530 (41.7%)
West	881 (17.3%)	1171 (19.3%)
Unknown	9 (0.2%)	18 (0.3%)
Insurance plan type, *N* (%)		
Medicare	639 (12.5%)	1562 (25.7%)
Commercial insurance	4468 (87.5%)	4510 (74.3%)
Year of index date		
2004	109 (2.1%)	264 (4.3%)
2005	276 (5.4%)	532 (8.8%)
2006	223 (4.4%)	427 (7.0%)
2007	288 (5.6%)	432 (7.1%)
2008	357 (7.0%)	479 (7.9%)
2009	400 (7.8%)	464 (7.6%)
2010	436 (8.5%)	454 (7.5%)
2011	386 (7.6%)	487 (8.0%)
2012	367 (7.2%)	460 (7.6%)
2013	406 (7.9%)	473 (7.8%)
2014	450 (8.8%)	467 (7.7%)
2015	597 (11.7%)	490 (8.1%)
2016	609 (11.9%)	447 (7.4%)
2017	203 (4.0%)	196 (3.2%)
Time from first observed CD diagnosis to index date, months, mean ± SD	28.2 ± 24.7	23.6 ± 23.0
Claim-derived moderate-to-severe patient status^a^, *N* (%)	3313 (64.9%)	2406 (39.6%)
Quan-CCI, mean ± SD	0.75 ± 1.3	1.0 ± 1.7
IBD-related surgeries, *N* (%)	418 (8.2%)	373 (6.1%)
Comorbidities (top 5), *N* (%)		
Pain	2990 (58.5%)	3162 (52.1%)
Cardiovascular disease	2323 (45.5%)	3212 (52.9%)
Anemia	1767 (34.6%)	1793 (29.5%)
Fatigue	1093 (21.4%)	1272 (20.9%)
Depression	876 (17.2%)	938 (15.4%)
Medication use		
Corticosteroids	3688 (72.2%)	3395 (55.9%)
Opioids	3055 (59.8%)	3235 (53.3%)
5-ASA	2311 (45.3%)	1738 (28.6%)
Immunomodulators	2212 (43.3%)	1680 (27.7%)
Antibiotics	2005 (39.3%)	645 (10.6%)
Biologics (other than index agent)	953 (18.7%)	—^b^
Healthcare costs (USD 2019), mean ± SD	34 084 ± 38 697	25 130 ± 53 494
Prescription drug costs (including biologic costs)	7407 ± 12 121	3689 ± 7035
Total medical costs (including biologic costs)	26 677 ± 36 350	21 441 ± 52 310

Abbreviations: 5-ASA, 5-aminosalicylic acid; CD, Crohn’s disease; IBD, inflammatory bowel disease; Quan-CCI, Quan-Charlson Comorbidity Index; SD, standard deviation.

Claim-derived moderate-to-severe patients defined as patients with any of the following indicator(s): patients receiving biologics, patients receiving immunomodulators, patients receiving corticosteroids (≥90 days of continuous use), patients with IBD-related hospitalizations (including IBD-related surgeries).

Per design, patients in the conventional cohort could not use biologics during their baseline.

#### Conventional cohort

In the conventional cohort, a total of 6072 CD patients initiated on a conventional agent were included and had a mean age of 50.5 years with 59.4% female (see Table 2). The most common index agent mechanism of action was 5-ASAs (62.9%) with 48.3% on mesalamine, 10.0% on sulfasalazine, 4.5% on balsalazide, and 0.1% on olsalazine. Among patients using immunomodulators (37.1%), the most common agent was azathioprine (19.3%) followed by mercaptopurine (13.0%), methotrexate (3.7%), with the remaining patients on mycophenolate, tacrolimus, cyclosporine, leflunomide, and thalidomide. The mean Quan-CCI index was 1.0, 39.6% of patients were considered to have claim-derived moderate-to-severe CD, and 55.9% of patients had ≥1 claim for corticosteroids. Finally, the mean baseline PPPY healthcare costs were $25 130, with the majority of the costs ($21 441) being medical.

### Indicators of Suboptimal Treatment

#### Biologic cohort

**Figure 1.**
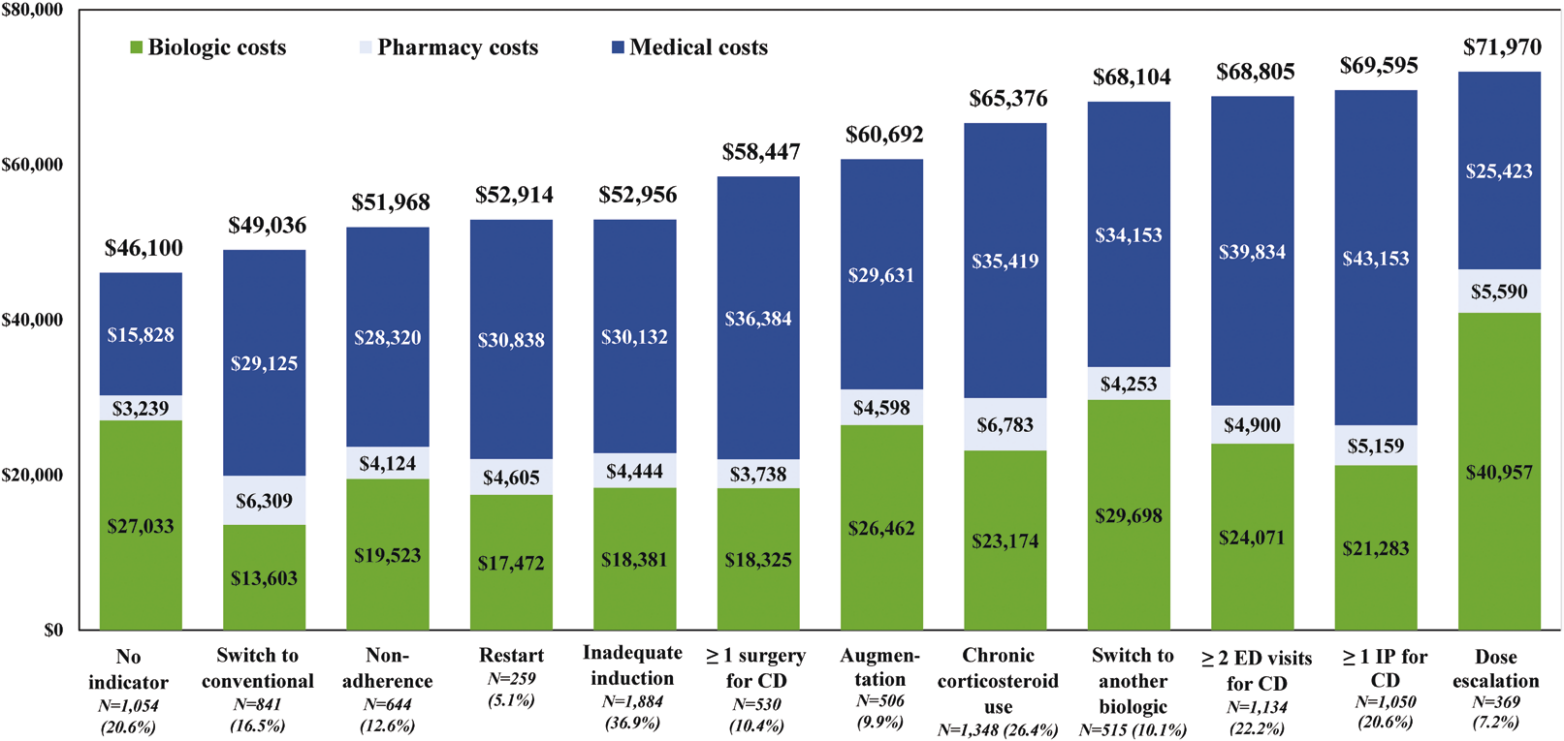
Healthcare costs per-patient-per-year (USD 2019) per indicator of suboptimal treatment subgroup during the 12 months following the landmark period for patients with CD using biologics during the landmark period (*N* = 5107) (biologic costs included the injection costs from the medical claims and the medication costs from the pharmacy claims; pharmacy costs excluded biologic costs; medical costs excluded biologic costs). Abbreviations: CD, Crohn’s disease; ED, emergency department; IP, inpatient; USD, United States dollars.

**Figure 2.**
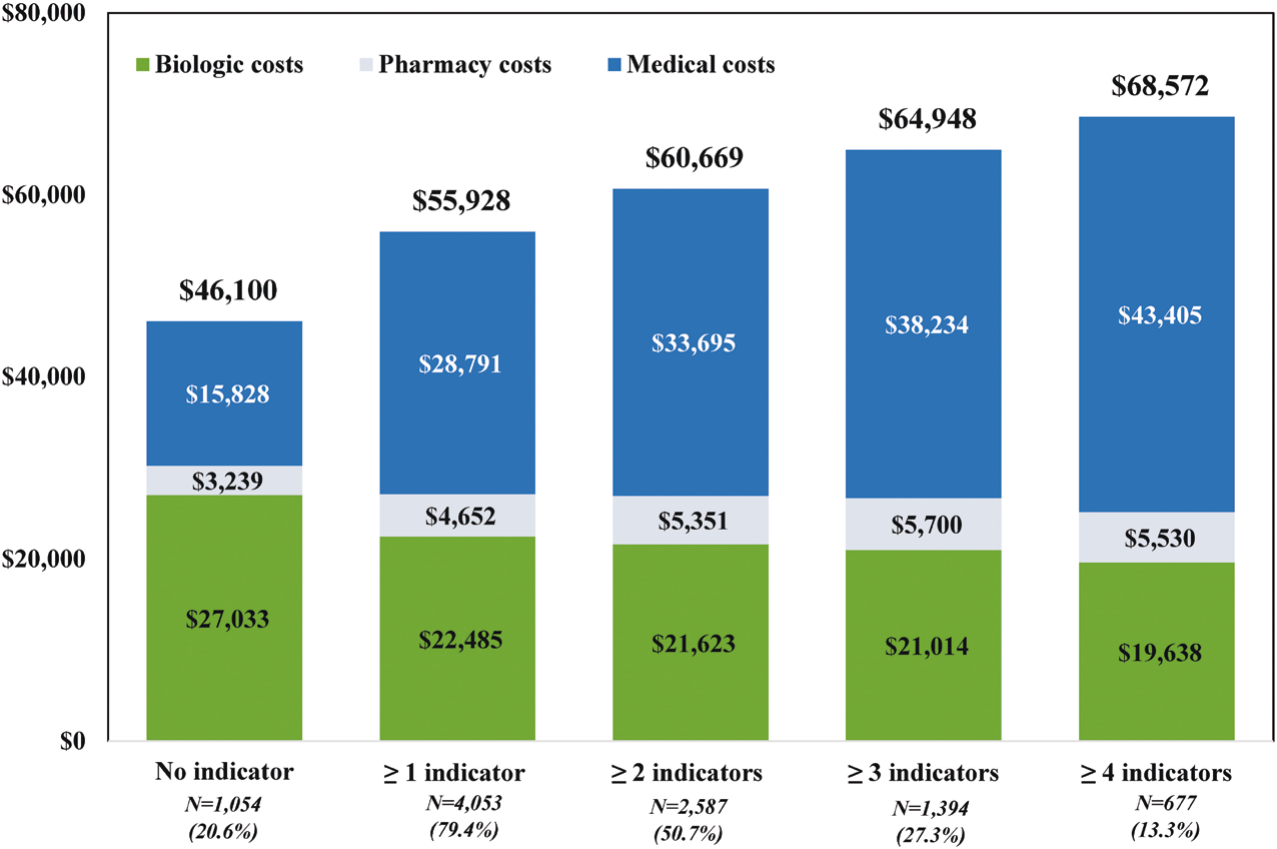
Healthcare costs per-patient-per-year (USD 2019) per number of suboptimal treatment indicator(s) during the 12 months following the landmark period for patients with CD using biologics during the landmark period (*N* = 5107) (biologic costs included the injection costs from the medical claims and the medication costs from the pharmacy claims; pharmacy costs excluded biologic costs; medical costs excluded biologic costs). Abbreviations: CD, Crohn’s disease; ED, emergency department; USD, United States dollars.

In the biologic cohort, there were 1054 patients (20.6%) without any indicator of suboptimal treatment, and 4053 patients (79.4%) with ≥1 indicator of suboptimal treatment during the 12-month landmark period following index. The most common indicator was inadequate biologic induction dose (36.9%), followed by chronic corticosteroid use (26.4%), and having ≥2 ED visits for CD (22.2%). There were 677 patients (13.3%) patients with ≥4 indicators of suboptimal treatment (see Figures 1 and 2).

#### Conventional cohort

**Figure 3.**
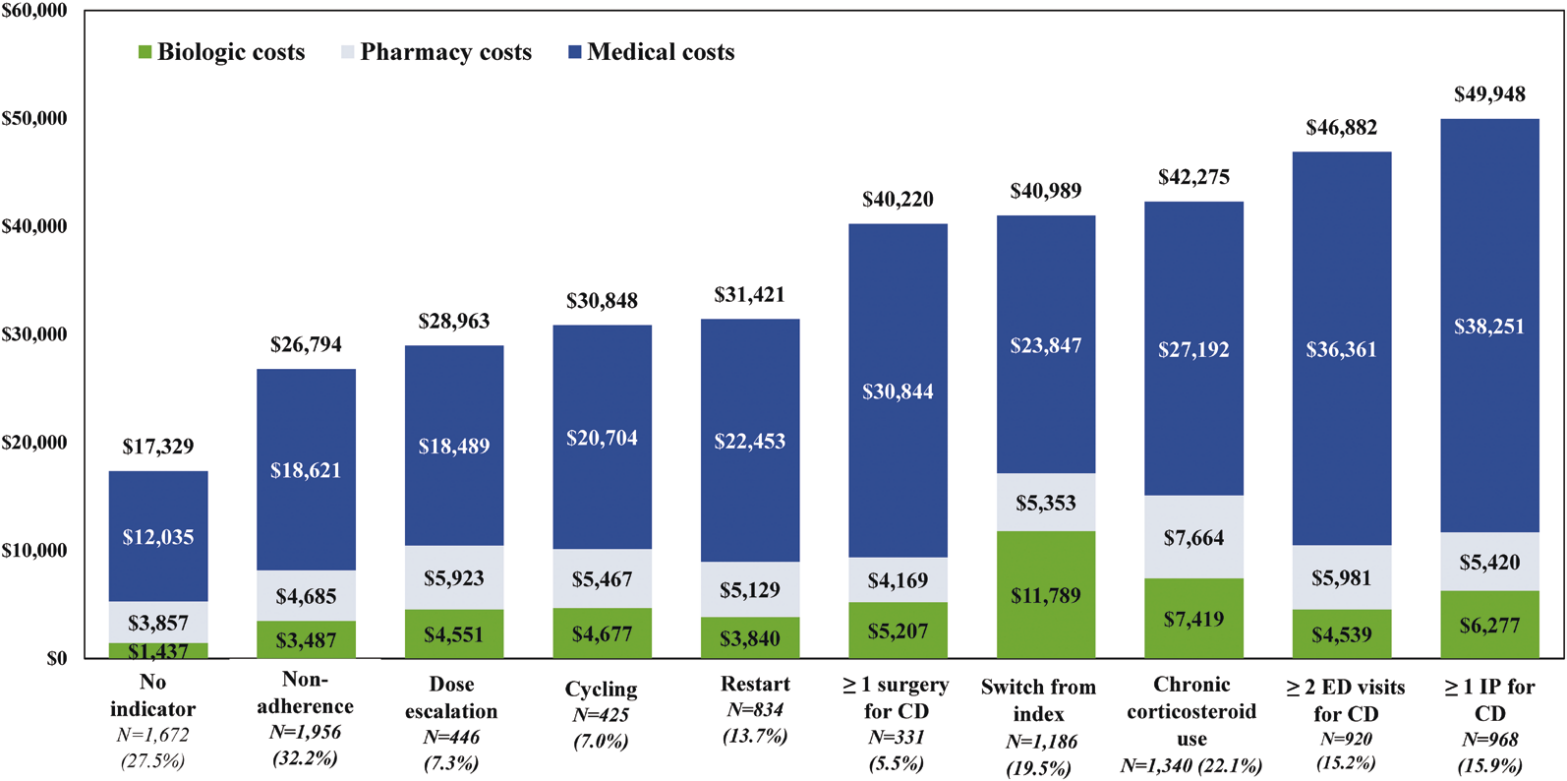
Healthcare costs per-patient-per-year (USD 2019) per indicator of suboptimal treatment subgroup during the 12 months following the landmark period for patients with CD using conventional agents during the landmark period (*N* = 6072) (biologic costs included the injection costs from the medical claims and the medication costs from the pharmacy claims; pharmacy costs excluded biologic costs; medical costs excluded biologic costs; only suboptimal treatment subgroup representing more than >5% of patients are shown, see Supplementary Table S3 for details). Abbreviations: CD, Crohn’s disease; ED, emergency department; IP, inpatient; USD, United States dollars.

**Figure 4.**
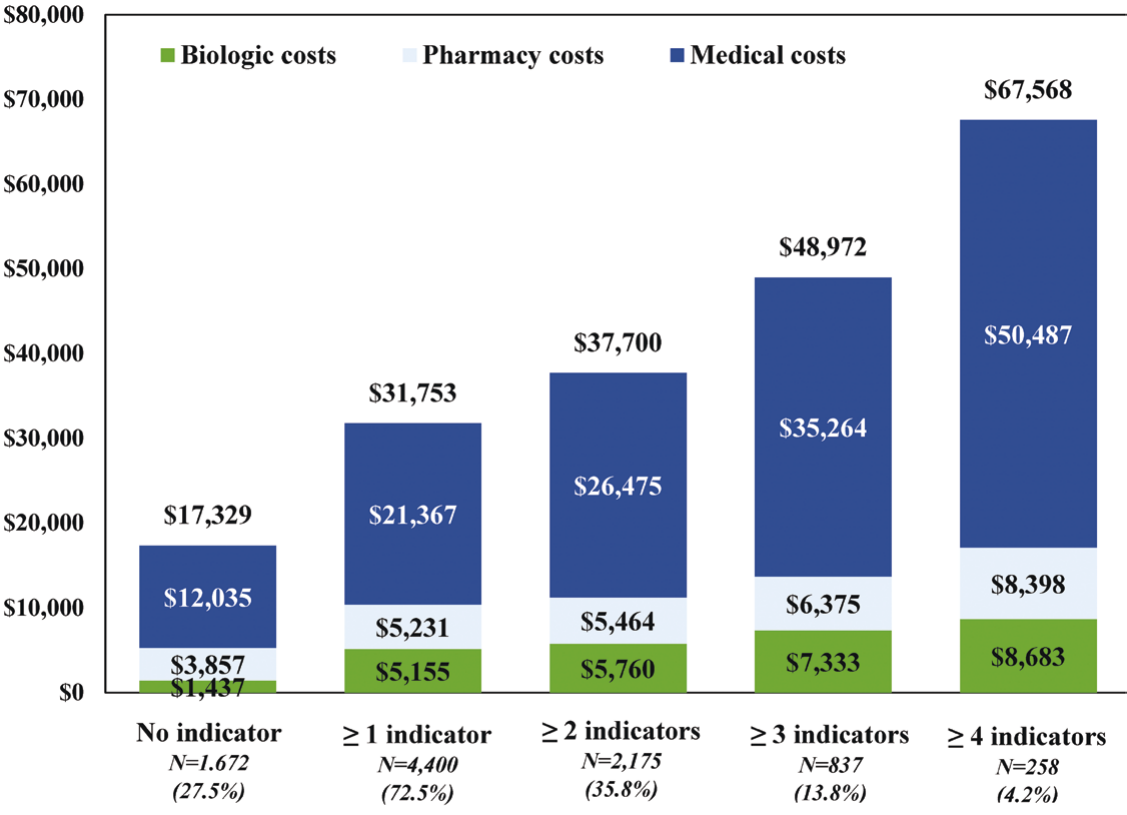
Healthcare costs per-patient-per-year (USD 2019) per number of suboptimal treatment indicator(s) during the 12 months following the landmark period for patients with CD using conventional agents during the landmark period (*N* = 6072) (biologic costs included the injection costs from the medical claims and the medication costs from the pharmacy claims; pharmacy costs excluded biologic costs; medical costs excluded biologic costs). Abbreviations: CD, Crohn’s disease; ED, emergency department; USD, United States dollars.

In the conventional cohort, out of 6072 patients included in the sample, 1672 patients (27.5%) did not have any indicator of suboptimal treatment, while 4400 patients (72.5%) had ≥1 indicator and 4.2% of patients had ≥4 indicators of suboptimal treatment during the landmark period. The most common indicators were: nonadherence to index treatment (32.2%), chronic corticosteroid use (22.1%), and switching from index treatment to a biologic or to a conventional agent with a different mechanism of action (19.5%) (see Figures 3 and 4).

### Healthcare Costs per Subgroup

#### Biologic cohort

The mean total PPPY healthcare costs varied across subgroups of patients with different indicators, and ranged from $49 036 for patients switching to a conventional treatment up to $71 970 for patients with a dose escalation, while the mean total PPPY healthcare costs was $46 100 for patients without indicator of suboptimal treatment. The increase in the mean PPPY healthcare costs was mostly driven by increases in the mean PPPY medical costs (eg, IP, ED, and OP excluding biologics costs) which varied from $25 423 in the dose escalation subgroup to $43 153 in the subgroup with ≥1 IP for CD, while the subgroup without an indicator had a cost of $15 828 (see Figure 1 and Supplementary Table S1).

Independently from the indicator, an increase in the number of suboptimal treatment indicators was numerically associated with an increase in the mean PPPY total healthcare costs from $55 928 (≥1 indicator) to $68 572 (≥4 indicators). The mean total biologic cost decreased as the number of suboptimal treatment indicators increased from $22 845 (≥1 indicator) to $19 638 (≥4 indicators), while the mean total biologic cost was $27 033 for the no indicator subgroup. The mean total healthcare cost increase was mostly driven by increasing medical costs which ranged from $28 791 (≥1 indicator) to $43 405 (≥4 indicators; see Figure 2 and Supplementary Table S2).

#### Conventional cohort

The mean total PPPY healthcare costs ranged from $26 794 to $53 842 for patients with a suboptimal treatment indicator, and patients with none of the suboptimal treatment indicators had a mean total PPPY of $17 329. The mean medical costs PPPY (notably IP and OP costs) ranged from $18 489 in the dose escalation subgroup to $38 251 in the ≥1 IP for CD subgroup, while the cost was $12 035 in the subgroup without an indicator The medical costs were the main contributors to the observed increase in the mean total PPPY healthcare costs. Increases in the mean total biologic costs PPPY in the subgroups with an indicator were also observed, with the largest increase occurring in the augmentation subgroup ($23 736; see Figure 3 and Supplementary Table S3).

Independently from the indicator, the mean total PPPY healthcare costs increased numerically as the number of suboptimal treatment indicators increased from $31 753 (≥1 indicator) to $67 568 (≥4 indicators). All categories of mean costs PPPY increased numerically with the number of suboptimal treatment indicators; the most important increase occurred for the medical costs that increased from $21 367 (≥1 indicator) to $50 487 (≥4 indicators; see Figure 4 and Supplementary Table S4).

## Discussion

In this study, using real-world data from a large population of patients with CD, 79.4% and 72.5% of the patients in the biologic and conventional cohorts, respectively, presented at least 1 indicator of suboptimal treatment within 1 year after treatment initiation. When the number of suboptimal treatment indicators observed was higher, the total healthcare costs PPPY in the following year tended to also be higher. Descriptively speaking, relative to patients with no suboptimal treatment indicators, the subgroup of patients with at least 4 indicators was associated with mean healthcare costs that were $22 472 higher ($46 100 vs $68 572) in the biologic cohort, and $50 239 higher ($17 329 vs $67 568) in the conventional cohort. While biologics contributed to a portion of the increase in costs related to suboptimal treatment, the pattern was mostly driven by medical costs (IP, ED, and OP excluding biologics) in both cohorts, and suggests an increasing burden to the healthcare system associated with suboptimal treatment.

Indeed, CD is associated with a significant burden to the healthcare system relative to patients without CD.^2–5^ However, there is a range of healthcare costs among CD patients, with concomitant variation in the disease burden. For instance, in a study using healthcare claims from 1999 to 2017 CD patients with a surgery presented an annual healthcare cost of $101 013, a value numerically higher than the general CD population ($24 500).^2^ The current study adds to the knowledge regarding the burden of CD, and presents the economic costs to the healthcare system in diverse subgroups of patients with suboptimal treatment indicator.

The pattern of increasing burden to the healthcare system among patients with a suboptimal treatment indicator has been reported in preceding studies typically focusing on adherence. Carter et al using retrospective claims from 2004 to 2009 reported that 77% of patients were adherent to infliximab, and significantly greater hospital costs were observed among nonadherent patients ($40 822) compared to adherent patients ($13 704).^20^ Similarly, Feagan et al focused on adherence to infliximab and reported higher medical costs in intermittently adherent ($20 068) compared to adherent patients ($13 097) using retrospective claims data from 2005 to 2010.^21^ Finally, Govani et al reported that poor adherence was associated with a 50% increase in hospitalization and steroid use in an insurance claims study.^22^

Rubin et al, assessing a range of suboptimal indicators in addition to adherence using commercial insurance claims data from 2006 to 2010, reported that approximately 80% of patients with CD had ≥1 indicator of suboptimal therapy, and that those patients had higher healthcare costs ($18 736) compared to patients without any indicator ($10 877 in 2011 USD)^14^ Other studies have reported that approximately 85% of patients with CD experienced ≥1 indicator of suboptimal biologic therapy within 2 years of initiating biologics therapy, but without quantifying the associated healthcare costs.^23,24^ In the current study, the proportion of patients with ≥1 indicator of suboptimal therapy ranged 73%–79%, which is comparable to the values reported in prior studies. Moreover, the difference in costs between patient without and with ≥1 indicator of suboptimal in this study ($9828 in the biologic cohort and $14 424 in the conventional cohort) was within range of Rubin et al after adjusting for inflation.^14^

In addition to confirming prior results with data extending to 2019 and encompassing a diversity of index agents and suboptimal indicators, this study also evaluated how common each suboptimal treatment was and their associated healthcare costs. Some new findings were revealed in the study. In the biologic cohort, switching to a conventional agent led to a reduction in biologics cost but an increase in medical costs so that the total healthcare costs PPPY was $49 036 in that subgroup, while it was $46 100 in the no indicator subgroup. That pattern, of a decrease in biologics cost and an increase in medical costs, was similar in the nonadherent and restart subgroups. Moreover, 26.4% and 22.1% of CD patients had chronic corticosteroid use in the biologic and conventional cohorts, respectively. The associated costs in the chronic corticosteroid subgroup were higher than in the no indicator subgroup suggesting a high burden in that subgroup. The total healthcare costs PPPY for chronic corticosteroid use in the conventional cohort was $42 275, only slightly and numerically lower than that in the no suboptimal indicator subgroup of the biologic cohorts ($46 100).

The study results also show that the healthcare costs tended to be numerically lower in the conventional cohort relative to the biologic cohort. However, in both cohorts the subgroups with ≥2 ED visits or ≥1 IP stay for CD were among the most expensive. In addition, in both cohorts the total healthcare costs PPPY were similar in the subgroup with ≥ 4 indicators, reaching $68 572 and $67 568 in the biologic and conventional cohorts, respectively.

Although this study focused on healthcare costs, suboptimal treatment may also impact patients’ quality-of-life. Indeed, the increased healthcare medical costs signify more frequent OP or ED visits and IP stays for the patient. In addition, prior research has suggested that nonadherence was associated with increased disability, including difficulty managing bowel movements, rectal bleeding, and arthralgia/arthritis.^25^ Conversely, better drug compliance was associated with higher quality-of-life based on the Crohn’s and Ulcerative Colitis Questionnaire (CUCQ-8) in another study.^26^ Further research is necessary to provide a comprehensive assessment of the impact of suboptimal treatment on patients with CD.

The current study may also have policy implications. The descriptive analysis presents indirect evidence that a step-up approach, where patients are initiated on less expensive conventional therapies before switching to biologics (if necessary), may not necessarily reduce the burden of CD on the healthcare system.^27,28^ Indeed, patients on conventional therapy with several indicators of suboptimal therapy had numerically higher medical costs relative to patients treated with biologics that had no or few indicators of suboptimal therapy, which suggests a substantial burden while the conventional therapy was being optimized. This further suggests that a top-down approach, where patients are treated with biologics early in the disease course to improve outcomes and prevent progression to irreversible bowel damage, may also reduce the burden of CD on the healthcare system.^29,30^ Studies evaluating clinical and economic outcomes for different treatment sequences are warranted to assess step-up or top-down approaches, and may also help target patients who would benefit most from early biologic treatment.^31^

### Limitations

This study is subject to several limitations. First, regarding the study design and database, the scope of the study was retrospective and descriptive. There were no adjustments for baseline confounders among the subgroups, and differences between the subgroups can be interpreted as associations. Moreover, as in any claims database, there may have been coding inaccuracies, and a recorded prescription fill did not imply that the medication was taken as prescribed. Finally, the claims database did not have certain clinical variables (eg, severity based on clinical criteria) and patient characteristics (eg, weight) to provide context to the analysis; notably, some dose escalation may be due to an increase in weight. Further, the claim-based definition of inadequate dose induction used in this study is not validated. Second, regarding the outcomes, the reasons leading to the observed suboptimal treatment indicator could not be evaluated. For instance, a discontinuation of index agent may be caused by a suboptimal event (clinical symptom, nonresponse, adverse event) but also a lack of access due to insurance reasons unrelated to a suboptimal event. Moreover, withdrawal of maintenance immunomodulator or biological agent can occur for low-risk patients who have been in deep remission for 2–4 years.^32^ Such “drug holidays” do not necessarily signal a suboptimal treatment event. To mitigate these problems, discontinuation alone was not considered a suboptimal indicator. Rather, restarts, switches, and cycling following discontinuation were considered suboptimal indicators if these events occurred within 12 months of initiation, before a drug holiday is warranted. Moreover, to reduce the probability of including patients without any suboptimal events, the healthcare costs outcomes were also provided for patients with several suboptimal indicators. Third, regarding the study sample, the study required the selection of patients with at least 3 years of continuous healthcare insurance eligibility. This may result in an underestimation of the effects of suboptimal treatment on outcomes as only well-insured patients were included. Finally, results may not be generalizable to the population of CD patients at large given specific study criteria among a privately insured patient population.

## Conclusions

In this retrospective study covering 15 years and thousands of patients, most CD patients treated with a biological or conventional agent (ie, 5-ASA and immunomodulators) presented indicators of suboptimal treatment which were associated with numerically higher healthcare costs than patients in the no suboptimal treatment indicator cohort. Notably, an increase in the number of suboptimal treatment indicators was associated with a numerical increase in the total healthcare costs. This pattern was mostly driven by increases in the IP and OP medical costs (excluding biologics costs). The patterns observed in this descriptive analysis suggest that suboptimal treatment continues to be prevalent in CD patients, and indicators of suboptimal treatment are associated with an increased burden to the healthcare system.

## Supplementary Material

otac021_suppl_Supplementary_Material

## Data Availability

The data that support the findings of this study are available from Optum, but restrictions apply to the availability of these data, which were used under license for the current study, and so are not publicly available. Any researchers interested in obtaining the data used in this study can access the database through Optum, under a license agreement, including the payment of appropriate license fee.
