# Supplementary material for: Indicators of Suboptimal Treatment and Associated Healthcare Costs Among Patients With Crohn’s Disease Initiated on Biologic or Conventional Agents
Source: Crohns Colitis 360. 2022 Jun 16;4(3):otac021. doi: 10.1093/crocol/otac021 (PMC9802278; doi:10.1093/crocol/otac021)
Supplement: otac021_suppl_Supplementary_Material [file otac021_suppl_supplementary_material.docx]

# **SUPPLEMENTARY INFORMATION**

**Figure S1. Study design**


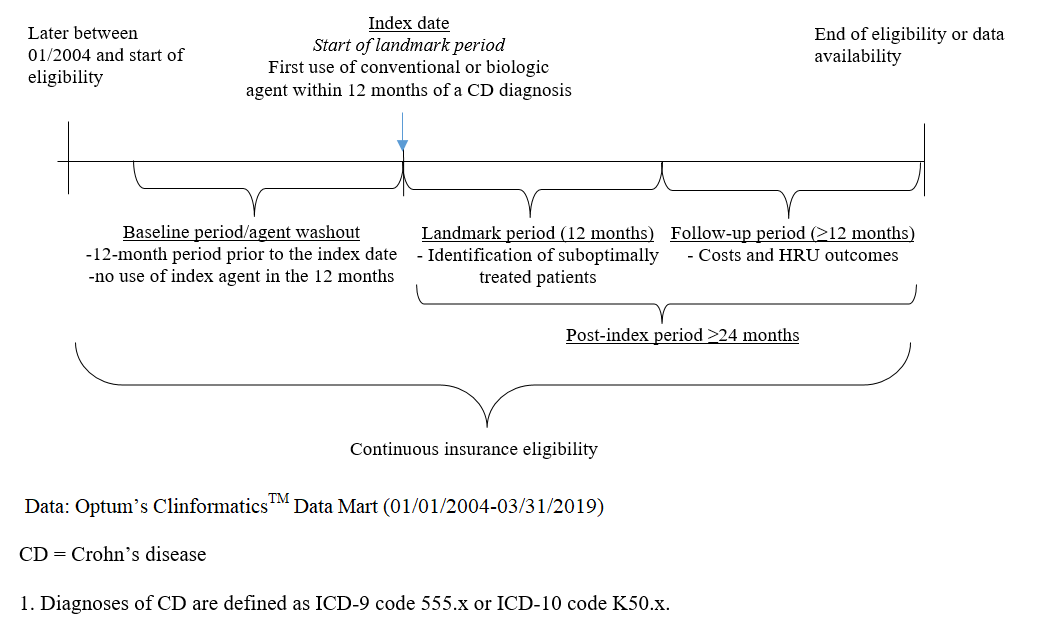


**Table S1.** **Healthcare costs per-patient-per-year (USD 2019) per indicator of suboptimal treatment subgroup during the 12 months following the landmark period for patients with CD using biologics during the landmark period: breakdown per setting (N=5,107)**

| ***Indicators in landmark period*** | ***No indicator*** | ***Switch to conventional*** | ***Non-adherence*** | ***Restart*** | ***Inadequate induction*** | ***≥ 1 surgery for CD*** | ***Augmen***  ***tation*** | ***Chronic cortico-***  ***steroid use*** | ***Switch to another biologic*** | ***≥ 2 ED visits for CD*** | ***≥ 1 IP for CD*** | ***Dose escalation*** |
| --- | --- | --- | --- | --- | --- | --- | --- | --- | --- | --- | --- | --- |
| **Costs (USD 2019) over 12 months after the landmark period** | **N=1,054**  **(20.6%)** | **N=841**  **(16.5%)** | **N=644**  **(12.6%)** | **N=259**  **(5.1%)** | **N=1,884**  **(36.9%)** | **N=530**  **(10.4%)** | **N=506**  **(9.9%)** | **N=1,348**  **(26.4%)** | **N=515**  **(10.1%)** | **N=1,134**  **(22.2%)** | **N=1,050**  **(20.6%)** | **N=369**  **(7.2%)** |
| Total costs, mean (SD) | 46,100 (39,291) | 49,036 (71,338) | 51,968 (56,455) | 52,914 (56,356) | 52,956 (58,740) | 58,447 (77,702) | 60,692 (61,850) | 65,376 (71,030) | 68,104 (73,987) | 68,805 (88,060) | 69,595 (93,118) | 71,970 (64,381) |
| Total biologic costs | 27,033 (21,713) | 13,603 (26,390) | 19,523 (24,595) | 17,472 (21,829) | 18,381 (24,734) | 18,325 (24,767) | 26,462 (26,294) | 23,174 (27,338) | 29,698 (33,796) | 24,071 (28,268) | 21,283 (27,764) | 40,957 (30,900) |
| Medical | 15,828 (30,002) | 29,125 (65,628) | 28,320 (50,593) | 30,838 (48,765) | 30,132 (53,876) | 36,384 (74,940) | 29,631 (58,525) | 35,419 (66,622) | 34,153 (68,757) | 39,834 (82,756) | 43,153 (88,370) | 25,423 (55,421) |
| Inpatient costs | 2,609 (12,337) | 7,444 (33,344) | 5,713 (17,146) | 5,007 (15,426) | 5,570 (20,967) | 9,387 (38,557) | 5,778 (35,305) | 7,961 (34,796) | 8,485 (38,558) | 8,664 (28,076) | 11,023 (35,462) | 7,161 (39,784) |
| ED costs | 830  (2,497) | 2,328 (7,578) | 2,564 (7,998) | 2,869 (6,848) | 2,138 (6,356) | 2,426 (7,078) | 1,637 (5,366) | 2,746 (9,602) | 2,884 (12,209) | 4,667 (12,510) | 4,082 (11,644) | 1,805 (5,762) |
| Outpatient costs | 11,223 (22,292) | 17,317 (38,127) | 17,807 (36,320) | 20,780 (36,285) | 20,633 (40,196) | 21,807 (46,736) | 20,648 (38,322) | 22,318 (42,033) | 20,385 (33,642) | 23,725 (62,353) | 24,768 (64,033) | 13,738 (21,017) |
| Other costs | 1,166 (6,500) | 2,036 (9,271) | 2,237 (9,453) | 2,181 (8,445) | 1,792 (6,796) | 2,764 (11,099) | 1,568 (6,579) | 2,394 (9,102) | 2,400 (9,959) | 2,777 (10,561) | 3,281 (11,634) | 2,718 (11,965) |
| Pharmacy | 3,239 (13,079) | 6,309 (12,642) | 4,124 (7,482) | 4,605 (10,265) | 4,444 (8,355) | 3,738 (8,377) | 4,598 (12,577) | 6,783 (10,802) | 4,253 (6,455) | 4,900 (12,465) | 5,159 (12,257) | 5,590 (15,927) |

CD: Crohn’s disease; ED: emergency department; IP: inpatient; SD: standard deviation

**Note:** Biologic costs included the injection costs from the medical claims and the medication costs from the pharmacy claims; pharmacy costs excluded biologic costs; medical costs excluded biologic costs

**Table S2.** **Healthcare costs per-patient-per-year (USD 2019) per number of suboptimal treatment indicator during the 12 months following the landmark period for patients with CD using biologics during the landmark period: breakdown per setting (N=5,107)**

| ***Indicators in landmark***  ***period*** | ***≥ 1 indicator*** | ***≥ 2 indicators*** | ***≥ 3 indicators*** | ***≥ 4 indicators*** |
| --- | --- | --- | --- | --- |
| **Costs (USD 2019) over 12 months**  **after the landmark period** | **N=4,053**  **(79.4%)** | **N=2,587**  **(50.7%)** | **N=1,394**  **(27.3%)** | **N=677**  **(13.3%)** |
| Total costs, mean (SD) | 55,928 (63,956) | 60,669 (75,082) | 64,948 (77,046) | 68,572 (83,303) |
| Biologic costs | 22,485 (26,356) | 21,623 (27,952) | 21,014 (28,729) | 19,638 (27,622) |
| Medical costs | 28,791 (59,051) | 33,695 (69,932) | 38,234 (70,801) | 43,405 (78,304) |
| Inpatient costs | 6,053 (25,656) | 7,596 (30,574) | 9,214 (36,376) | 10,369 (37,325) |
| ED costs | 2,221 (7,491) | 2,758 (8,693) | 3,363 (10,462) | 4,201 (13,042) |
| Outpatient costs | 18,557 (42,318) | 21,083 (50,159) | 22,978 (42,759) | 25,456 (48,138) |
| Other costs | 1,960 (8,317) | 2,258 (8,869) | 2,679 (10,113) | 3,378 (12,409) |
| Pharmacy costs | 4,652 (9,969) | 5,351 (11,537) | 5,700 (12,070) | 5,530 (10,037) |

CD: Crohn’s disease; ED: emergency department; SD: standard deviation

**Note:** Biologic costs included the injection costs from the medical claims and the medication costs from the pharmacy claims; pharmacy costs excluded biologic costs; medical costs excluded biologic costs

**Table S3.** **Healthcare costs per-patient-per-year (USD 2019) per indicator of suboptimal treatment subgroup during the 12 months following the landmark period for patients with CD using conventional agents during the landmark period: breakdown per setting (N=6,072)**

| ***Indicators in landmark period*** | ***No indicator*** | ***Non-adherence*** | ***Dose escalation*** | ***Cycling*** | ***Restart*** | ***≥ 1 surgery for CD*** | ***Switch from index*** | ***Chronic cortico-***  ***steroid use*** | ***≥ 2 ED visits for CD*** | ***≥ 1 IP for CD*** | ***Augmen-***  ***tation*** |
| --- | --- | --- | --- | --- | --- | --- | --- | --- | --- | --- | --- |
| **Costs (USD 2019) over 12 months**  **after the landmark period** | **N=1,672**  **(27.5%)** | **N=1,956**  **(32.2%)** | **N=446**  **(7.3%)** | **N=425**  **(7.0%)** | **N=834**  **(13.7%)** | **N=331**  **(5.5%)** | **N=1,186**  **(19.5%)** | **N=1,340**  **(22.1%)** | **N=920**  **(15.2%)** | **N=968**  **(15.9%)** | **N=244**  **(4.0%)** |
| Total costs, mean (SD) | 17,329 (36,723) | 26,794 (57,477) | 28,963 (44,331) | 30,848 (57,068) | 31,421 (78,217) | 40,220 (73,785) | 40,989 (55,823) | 42,275 (58,414) | 46,882 (92,289) | 49,948 (84,623) | 53,842 (51,504) |
| Total biologic costs | 1,437 (7,636) | 3,487 (12,247) | 4,551 (11,492) | 4,677 (11,638) | 3,840 (15,115) | 5,207 (12,977) | 11,789 (18,513) | 7,419 (16,732) | 4,539 (12,329) | 6,277 (16,392) | 23,736 (23,137) |
| Medical | 12,035 (34,379) | 18,621 (54,094) | 18,489 (40,862) | 20,704 (53,773) | 22,453 (74,849) | 30,844 (71,473) | 23,847 (51,410) | 27,192 (54,344) | 36,361 (89,783) | 38,251 (81,991) | 24,935 (48,096) |
| Inpatient costs | 2,567 (14,592) | 4,060 (21,660) | 4,219 (20,894) | 4,720 (20,748) | 5,384 (29,957) | 9,075 (34,250) | 6,086 (26,125) | 6,621 (25,982) | 8,364 (33,168) | 10,492 (32,978) | 5,507 (24,830) |
| ED costs | 838  (2,769) | 1,825 (5,862) | 1,422 (5,499) | 1,945 (14,895) | 2,155 (6,731) | 2,162 (6,276) | 2,218 (10,455) | 2,970 (11,613) | 5,067 (14,689) | 4,399 (13,621) | 1,804 (5,758) |
| Outpatient costs | 7,527 (26,296) | 11,438 (34,611) | 11,602 (24,881) | 12,085 (38,430) | 13,344 (47,122) | 17,757 (40,057) | 14,119 (29,971) | 15,968 (32,089) | 20,910 (62,146) | 20,941 (53,454) | 15,885 (27,577) |
| Other costs | 1,103 (5,063) | 1,298 (6,067) | 1,245 (4,563) | 1,955 (7,887) | 1,570 (7,774) | 1,850 (5,110) | 1,424 (5,180) | 1,632 (5,067) | 2,021 (6,395) | 2,419 (10,548) | 1,739 (6,964) |
| Pharmacy | 3,857 (6,040) | 4,685 (9,983) | 5,923 (10,709) | 5,467 (10,899) | 5,129 (11,724) | 4,169 (6,673) | 5,353 (12,667) | 7,664 (12,746) | 5,981 (14,387) | 5,420 (9,743) | 5,172 (9,542) |

CD: Crohn’s disease; ED: emergency department; IP: Inpatient; SD: standard deviation

**Note:** Biologic costs included the injection costs from the medical claims and the medication costs from the pharmacy claims; pharmacy costs excluded biologic costs; medical costs excluded biologic costs

**Table S4.** **Healthcare costs per-patient-per-year (USD 2019) per number of suboptimal treatment indicator during the 12 months following the landmark period for patients with CD using conventional agents during the landmark period: breakdown per setting (N=6,072)**

| ***Indicators in landmark period*** | ***≥ 1 indicator*** | ***≥ 2 indicators*** | ***≥ 3 indicators*** | ***≥ 4 indicators*** |
| --- | --- | --- | --- | --- |
| **Costs (USD 2019) over 12 months**  **after the landmark period** | **N=4,400**  **(72.5%)** | **N=2,175**  **(35.8%)** | **N=837**  **(13.8%)** | **N=258**  **(4.2%)** |
| Total costs, mean (SD) | 31,753 (55,829) | 37,700 (68,431) | 48,972 (85,943) | 67,568 (127,550) |
| Biologic costs | 5,155 (13,563) | 5,760 (15,264) | 7,333 (17,927) | 8,683 (21,393) |
| Medical costs | 21,367 (52,349) | 26,475 (65,269) | 35,264 (82,804) | 50,487 (125,508) |
| Inpatient costs | 4,897 (22,028) | 6,416 (27,268) | 9,008 (34,681) | 13,787 (48,038) |
| ED costs | 2,114 (7,798) | 2,803 (10,247) | 4,263 (14,398) | 6,277 (21,218) |
| Outpatient costs | 12,898 (34,756) | 15,582 (42,704) | 19,770 (50,557) | 27,350 (79,259) |
| Other costs | 1,459 (6,340) | 1,675 (6,438) | 2,222 (8,745) | 3,073 (10,340) |
| Pharmacy costs | 5,231 (10,884) | 5,464 (10,332) | 6,375 (12,241) | 8,398 (15,777) |

CD: Crohn’s disease; ED: emergency department; SD: standard deviation

**Note:** Biologic costs included the injection costs from the medical claims and the medication costs from the pharmacy claims; pharmacy costs excluded biologic costs; medical costs excluded biologic costs
